# 3D printing of liquid crystal elastomers-based actuator for an inchworm-inspired crawling soft robot

**DOI:** 10.3389/frobt.2022.889848

**Published:** 2022-08-10

**Authors:** Xiaowen Song, Weitian Zhang, Haoran Liu, Limeng Zhao, Qi Chen, Hongmiao Tian

**Affiliations:** Micro-and Nano-technology Research Center, State Key Laboratory for Manufacturing Systems Engineering, Xi’an Jiaotong University, Xi’an, China

**Keywords:** 3D printing, liquid crystal elastomers, soft robot, anisotropic friction, tilted fish-scale-like microstructures

## Abstract

Liquid crystal elastomers (LCEs) have shown great potential as soft actuating materials in soft robots, with large actuation strain and fast response speed. However, to achieve the unique features of actuation, the liquid crystal mesogens should be well aligned and permanently fixed by polymer networks, limiting their practical applications. The recent progress in the 3D printing technologies of LCEs overcame the shortcomings in conventional processing techniques. In this study, the relationship between the 3D printing parameters and the actuation performance of LCEs is studied in detail. Furthermore, a type of inchworm-inspired crawling soft robot based on a liquid crystal elastomeric actuator is demonstrated, coupled with tilted fish-scale-like microstructures with anisotropic friction as the foot for moving forwards. In addition, the anisotropic friction of inclined scales with different angles is measured to demonstrate the performance of anisotropic friction. Lastly, the kinematic performance of the inchworm-inspired robot is tested on different surfaces.

## Introduction

Compared with conventional rigid robots, soft robots are made from soft materials (He and Cai, 2021; Ilami et al., 2021; Xiong et al., 2021; Li et al., 2022), and they exhibit unique features such as compliance, unlimited degrees of freedom, large deformation, and safe interaction with human beings. They can pass through channels smaller than their size (Jing et al., 2020; Shih et al., 2020; Dou et al., 2021; Pal et al., 2021; Roels et al., 2022). Soft robots have attracted much interest due to their practical applications in fragile spaces, complex pathways, and human-robot-environment interaction (Shih et al., 2020; Yang et al., 2020; Zhao et al., 2021). Soft actuators are the components that transduce the input energy into deformation and drive the robot’s motion (Wan et al., 2012). Various soft actuators have been widely developed in past decades, including pneumatic actuators, hydraulic actuators, ion-exchange polymer metal composite materials (IPMCs), dielectric elastomer actuators (DEAs), hydrogels, liquid crystal elastomers (LCEs), and shape memory alloys (SMAs) (Acome et al., 2018; Li et al., 2018; Rich et al., 2018; Chen et al., 2019; Yang et al., 2019; Lee et al., 2020; Suzumori et al., 2020; Zhao et al., 2021). The LCEs are made of liquid crystal mesogens and polymer networks. They are characterized by elastic properties of polymer networks and anisotropic and stimuli-responsive properties of liquid crystal mesogens. The aligned liquid crystal mesogens can undergo nematic-to-isotropic phase transition under external stimuli such as heat, light, electricity, magnetic field, or humidity. A change in the molecular orientation upon phase transition can lead to a macroscopic shape change (Zeng et al., 2018; Yang et al., 2019; Shen et al., 2020; Xiao et al., 2020). It should be noted that this deformation is reversible. The LCE reverts to its original shape when the external stimulus is removed.

The LCEs have been programmed into various motion modes and used to drive the motion of soft robots (da Cunha et al., 2020; You et al., 2020). Cai et al. proposed a soft robot driven by LCE-carbon nanotube (CNT) composite film prepared by uniaxial stretching (Ahn et al., 2019). The composite film was prepared by a two-step polymerization strategy with the addition of CNTs. The constructed robot can achieve multi-modal motions of squeezing, jumping, and crawling when stimulated by light scanning. Xia et al. reported a completely untethered soft robot based on Joule-heated LCE that can achieve steering, load, and other functions through Bluetooth control (Boothby et al., 2022). Hu et al. designed a snake-mimic soft actuator with a double-layer LCE ribbon. When irradiated by a near-infrared (NIR) light with multiple switches, the soft robot moved forward through reversible shape deformation between the S-shape structure and reversed S-shape structure (Wang M. et al., 2020). Souren et al. fabricated a double-layer actuator with dual responsiveness to the environmental humidity and temperature changes by spraying LCs (liquid crystal) on a stretched polyamide 6 (PA6) substrate (Verpaalen et al., 2020).

The LCEs can be fabricated into desired shapes through one-step or two-step polymerization methods, usually obtained in films or sheets. It is relatively difficult to design the LCE structure with controlled 3D shapes and desired alignment patterns (Buguin et al., 2006; Yakacki et al., 2015; Zhang C. et al., 2019). Recently emerging 3D printing technologies of LCEs can simplify the manufacturing process with computer-aided design and effectively control the 3D shape and alignment patterns. Direct ink writing technique (DIW) is a type of 3D printing technology based on extrusion (Zhu et al., 2020). The viscoelastic ink is extruded from the nozzle and deposited onto a substrate. The DIW technique has been effectively employed for LCEs printing (Kotikian et al., 2018). The liquid crystal mesogens are aligned along the printing path under shearing action during extrusion and stretching action between the nozzle and the substrate during nozzle movement. After extrusion, the printed filaments are cured by UV illumination to obtain reversible actuation properties. Thus, the alignment patterns can be controlled by the designed printing pathways (Zhang Y. B. et al., 2019; Balani et al., 2021; Tagliaferri et al., 2021).

**FIGURE 1:**
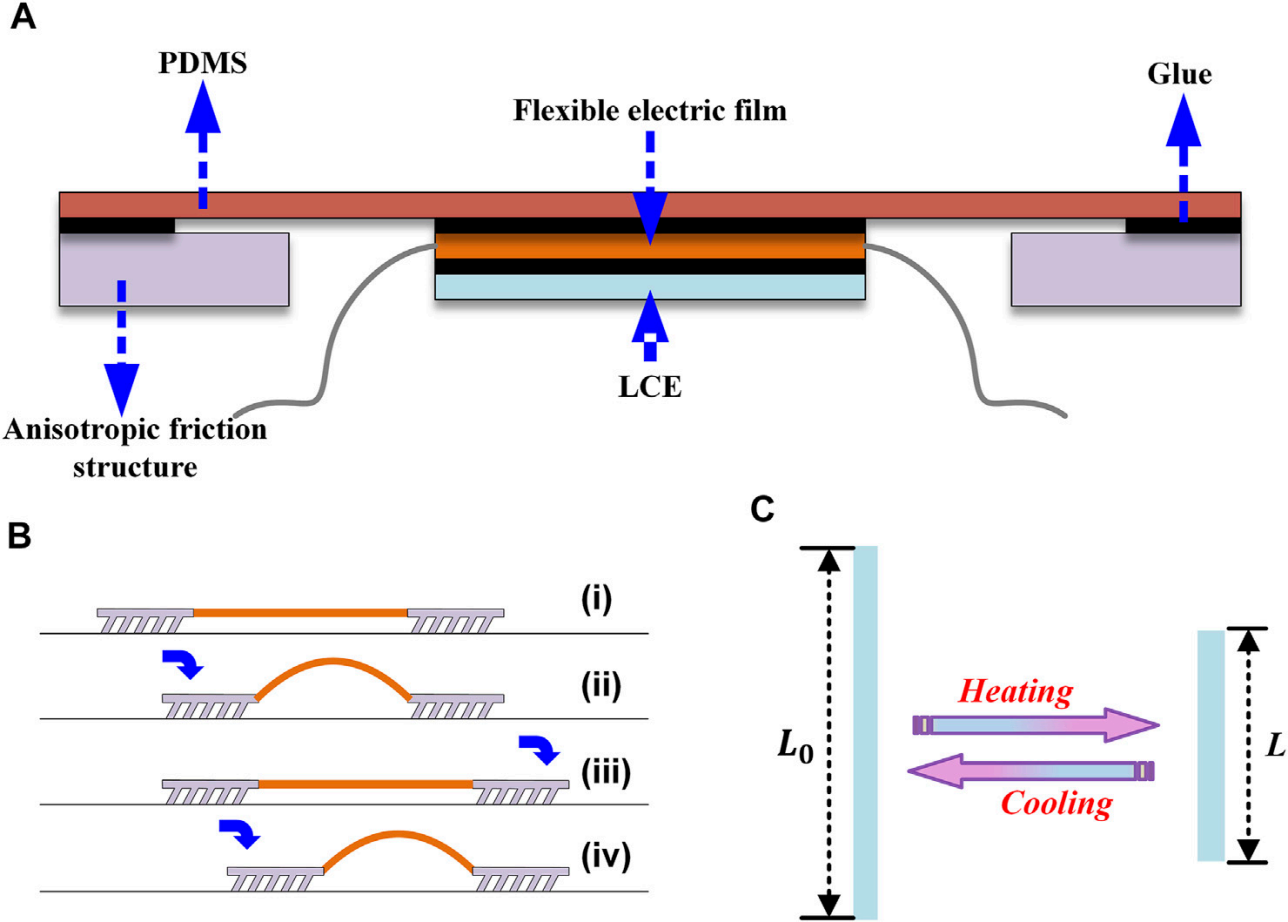
Schematic diagram of the structural design of the inchworm-inspired soft robot: **(A)** A schematic depiction of the robot’s overall construction. The actuating part is composed of LCE and flexible electrothermal film. The black part in the figure represents the glue bonding part. **(B)** Creep mechanism of an anisotropic friction structure. The robot can move forward when the actuation component is arched and stretched. **(C)** Thermal actuation principle of the LCE. When the temperature approaches the nematic-isotropic transition temperature, the elastomer will show a macroscopic change in length. When the temperature approaches the nematic-isotropic transition temperature, the length of the LCE rapidly shortens. The changing process is exactly the opposite when cooling from a high temperature.

However, the integration of LCEs printing technology with functional robotics has been rarely reported. In this study, the printing parameters in the DIW process are optimized to achieve optimal actuation performance of the printed LCEs, that is, larger actuation strain and stress. Furthermore, the printed LCE structure is utilized to construct an inchworm-inspired crawling soft robot. The inchworms can bend their bodies into an arch and then recover them into extended shapes. When the front and rear feet friction is not the same, the inchworms move forward. The inchworm-inspired robot consists of three parts: the flexible actuator, the front, and rear foot, and the connecting part between the two parts (Figure 1A). The foot is made from an anisotropic tilted fish-scale-like microstructure of Polydimethylsiloxane (PDMS) (Figure 1B), and the connection part adopts a soft PDMS. The flexible actuation part comprises an electrothermal film and a 3D-printed liquid crystal elastomer film (Figure 1C). When voltage is applied at both ends of the flexible electrothermal film, the film generates heat due to the Joule effect, and the LCE contracts. The flexible actuator bends into an arch due to the contraction mismatch between the LCE film and the electrothermal film. The anisotropic structures have more friction to the right than to the left. When the LCEs contract, the left foot and the arch shape of the main body move, as shown in Figure 1B, from (i) to (ii). When the external voltage is removed, the LCE film cools in the ambient environment and recovers to the initial length, creating an outward thrust on both feet. The friction force in the left foot is higher than the one in the right foot, thereby pushing the entire structure to the right [Figure 1B from (ii) to (iii)]. An optimized fish-scale-like microstructure with tilted angles following the work by Li et al. (2017) is prepared to introduce the anisotropic structure with appropriate friction. The fish-scale-like microstructures with different tilted angles can have different frictional forces in two directions, leading to the motion of the inchworm-like structure.

## Materials and methods

### Synthesis of LCE for 3D printing

The LCE ink is prepared following a previously reported formula through the Michael addition reaction of liquid crystal mesomorphic agent RM257 and chain extender 2,2'-(ethylenedioxy) diethanethiol (EDDET) (Wang Z. J. et al., 2020). First, RM257 (8.2404 g, 14 mmol) was dissolved in 50 ml dichloromethane. Then, difunctional thiol chain extender EDDET (2.1876 g, 12 mmol) and catalyst dipropylamine (0.100 g, 1 mmol) were added. After stirring at room temperature for 12 h, the photoinitiator Irgacure 2959 (0.0500 g, 0.2 mmol) was added, and the mixture was placed in an oven at 85°C for 24 h to remove the solvent.

### Fabrication of LCE-based soft actuator and anisotropic friction structure

**FIGURE 2:**
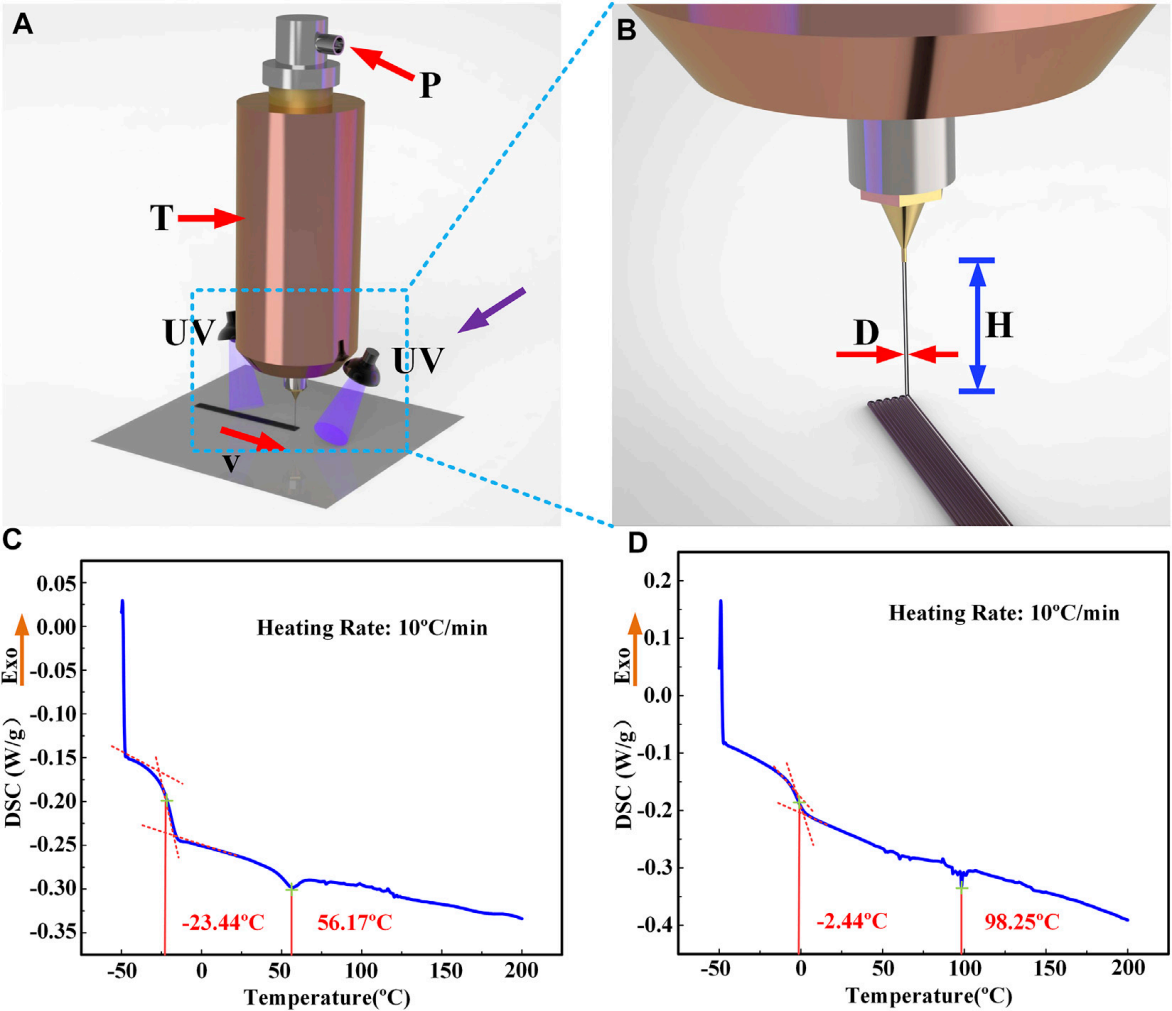
DIW printing process and printing parameters of LCE: the ink is made of an LC oligomer, which is extruded along the moving direction of the nozzle after being heated in the printer and simultaneously cured by UV. The involved process parameters include *v* (printing speed), *T* (printing temperature), *P* (extrusion pressure), *H* (printing height), and *D* (nozzle diameter). **(A)** The DIW macro printing process in its entirety. **(B)** The nozzle and printing materials are partially enlarged to demonstrate the extruded fiber and printing path. **(C)** DSC test of the LCE ink. **(D)** DSC test of cured LCE.

The LCE ink is printed by a DIW device, as shown in Figures 2A, B. The shape of the printed structure is programmed by employing computer-aided design. The LCE ink was loaded into a cylinder and heated to a temperature close to or higher than the nematic-isotropic transition temperature. Then, the LCE ink was extruded through a small nozzle. During extrusion, liquid crystal mesogens are aligned along the printing path due to the nozzle shearing and the tensile action between the nozzle and the substrate. The extruded LCE filaments were further crosslinked under 365 nm UV (ultraviolet) illumination irradiating to permanently fix the orientation. After crosslinking, the glass transition temperature and the nematic-isotropic phase transition temperature of LCE increased from −23.44 and 56.17 °C to −2.44 and 98.25°C, respectively, as determined by differential scanning calorimetry (DSC) (Figures 2C, D).

The fish-scale-like microstructures were fabricated by photolithography and molding processes. A mask with 5 μm × 25 μm rectangular micro-holes was used in the photolithography, and the inclined exposure was adopted to obtain the inclined microstructures. Furthermore, the PDMS (Dow Corning Sylgard 184), a commonly used soft material for micro/nano-imprint printing, was selected as the material to fabricate the inclined fish-scale-like microstructures.

The photoresist (AZ4620) was spun coated on a clean glass and placed on a heated plate at 95 °C for 10 min. The wavelength of the employed UV light source is G-line (436 nm), the power of the exposure light source is 12.5 mW·cm-2, and the exposure time is 150 s. The photoresist-coated glass and mask were clamped together and placed on an adjustable angle holder to control the exposure angle. After exposure, the sample was released from the mask and angle holder and immersed in a developing solution. After development, the PDMS was (the ratio of PDMS prepolymer and curing agent was 10:1) poured onto the patterned photoresist and placed in the oven at 80°C for 1 h. Once the PDMS was cured, the photoresist was sacrificed via acetone, resulting in the tilted fish-scale-like microstructures.

### Device integration

The robot is made of an actuatable main body, feet at both ends, and the connecting part in the middle. The actuating part of the main body consists of the active LCE layer and an electrothermal layer. The electrothermal film was prepared by scraping multi-walled carbon nanotubes (MWCNTs) onto a polyimide (PI) film and sintering after drying. The parameters of MWCNTs are shown in Table 1. The flexible actuator was connected to a power source with two copper wires, glued to the electrothermal film with conductive silver adhesive. The fish-scale-like microstructures with a 45° tilting angle made up of PDMS were employed for the feet. The actuating part and the feet were connected through a soft PDMS strip sheet with a thickness of 1 mm. For the connecting part, Dow Corning Sylgard 184 (with the ratio of the precursor to the curing agent of 10:1) was coated between two 1 mm-thick glass slides on a PET film. And then they were covered with another PET and pinned down with a thick glass plate. The thick glass plate was used to flatten PDMS and ensure that the thickness of PDMS was the same as that of the glass slide. Then, they were placed in the oven for curing.

**TABLE 1:** Parameters of MWCNTs.

Parameter	Range
Purity	>90 wt%
Inner Diameter	20–30 nm
Outer Diameter	50 nm
Length	10–30 μm
Specific Surface Area	>300 m^2^/g
Packing Density	0.18 g/cm^3^

The electrothermal film, PDMS, and inclined microstructures were cut to the same width, and the length of the PDMS strip was cut to at least 1–2 cm above the sum of the lengths of electrothermal film and two inclined scale structures. An organic silicone glue (the glue needs to be cured at room temperature for about 2 h) was then applied to the middle of the PDMS strip with the length of the electrothermal film to stick it on. The two ends of the PDMS strip were glued approximately half the length of the inclined scale structure. Then, the fish-scale-like microstructures were glued on. It should be noted that the inclined fish-scale-like microstructures of the feet at both ends were arranged in the same inclined direction. Due to flexible actuator bending, the inclined scale structure may be detached from the ground, leading to anisotropic friction failure. The PDMS length is additionally set aside and coated with half the length of glue to reduce the influence of actuator bending on anisotropic friction. Finally, the LCE was cut to the same length as the electrothermal film and glued together (organic silicone glue is used for all the aforementioned processes ).

## Results

### Effect of printing process parameters on properties of LCE

**FIGURE 3:**
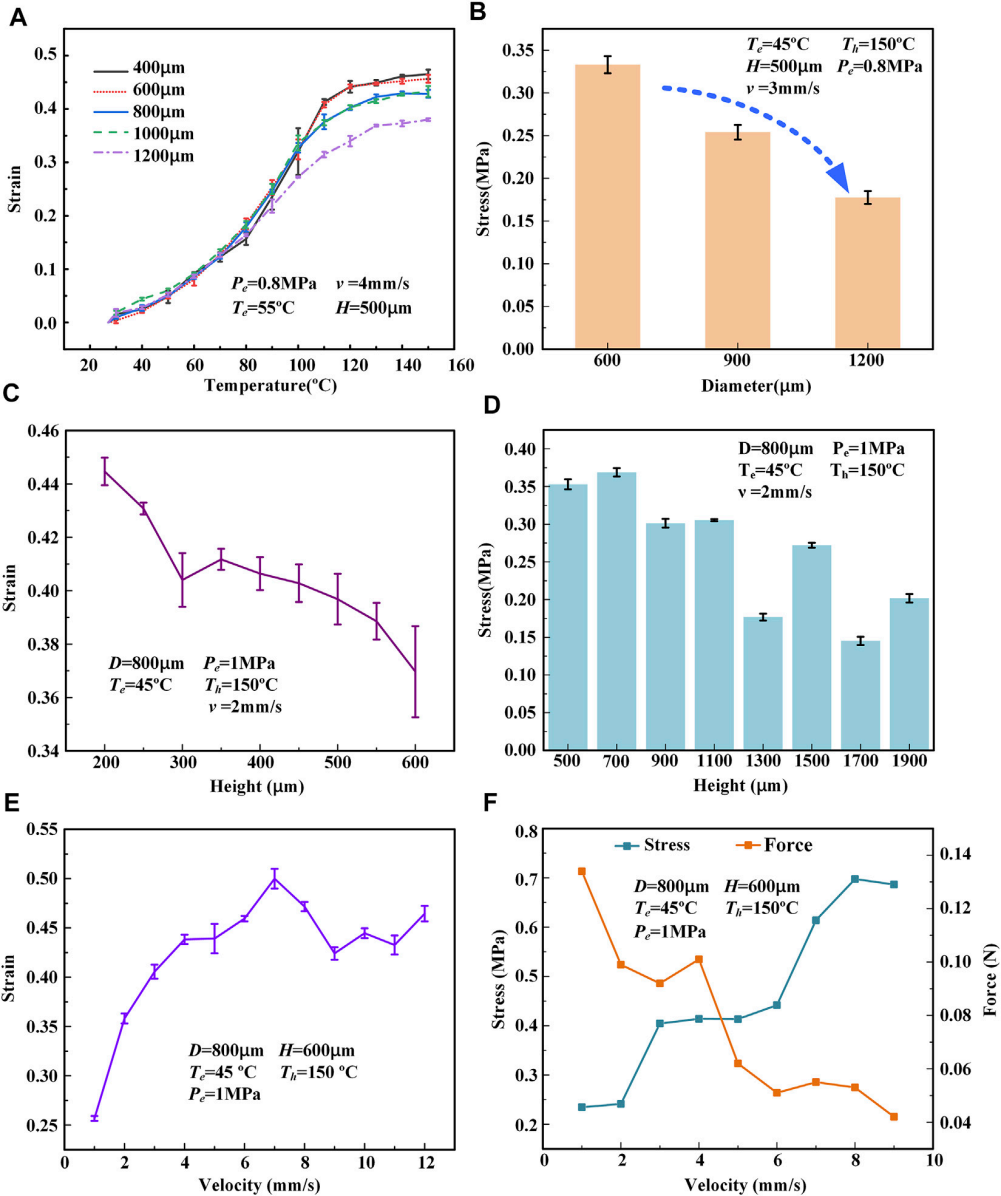
Actuation performance of printed LCE filaments under various printing parameters ( $T_{e}$ stands for the printing temperature at which the LCE is heated in the barrel during printing, $T_{f}$ indicates the temperature at which the printed LCE is heated, and $P_{e}$ is the extrusion pressure.) **(A)** The relationship between the actuation strain of a printed LCE fiber (when heated to different set temperatures) and the nozzle diameter *D* **(B)** The relationship between the actuation stress of the LCE fiber (when heated to 150 °C) and nozzle diameter *D* **(C)** The relationship between the actuation strain of printed LCE fiber (when heated to 150°C) and the printing height *H* **(D)** The relationship between the actuation stress of the LCE fiber (when heated to 150°C) and printing height *H* **(E)** The relationship between the actuation strain of the printed LCE fiber (when heated to 150°C) and printing speed *v.* **(F)** The relationship between the actuation stress and the actuation force (when heated to 150°C) of the printed LCE fiber and printing speed *v.*

**FIGURE 4:**
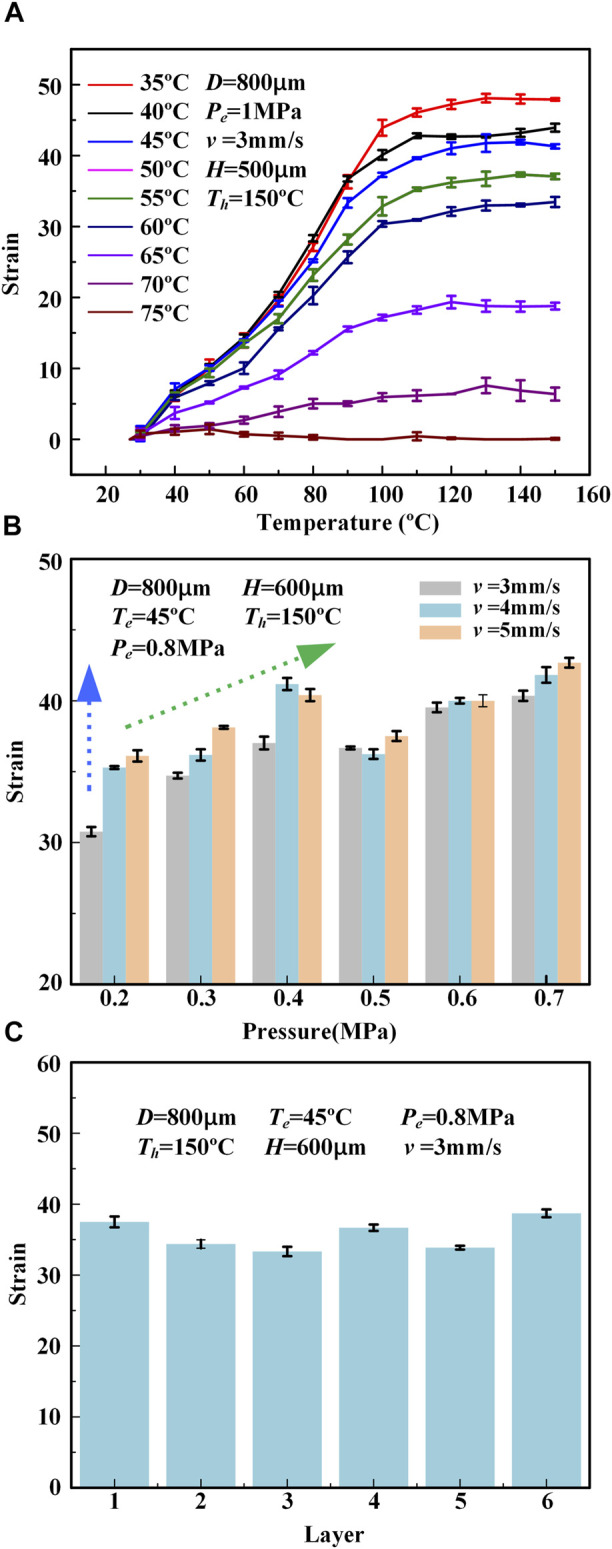
**(A)** The relationship between the actuation strain of the printed LCE fiber (when heated to different set temperatures) and printing temperature *T.* **(B)** The relationship between the actuation strain of the printed LCE fiber (when heated to 150°C) and extrusion pressure *P.* **(C)** The relationship between the actuation strain of the printed LCE sheet (when heated to 150°C) and the number of printed layers.

To characterize the 3D printed LCE actuating performance, the actuation strain and actuation stress of the printed LCEs were carefully tested under different printing parameters, including the printing speed *v*, nozzle diameter *D*, printing height *H* (distance between print heads and substrate), printing temperature *T* (the temperature at which LCE ink is heated in the barrel), extrusion pressure *P* (the pressure provided by air pressure source), and printing layers (layers of LCE flat film along the vertical direction), as shown in Figure 2. The actuation strain *ε* is calculated as $\varepsilon \;=\left(L_{0}-L\right)/L_{0}$ , where $L_{0}$ stands for the original length of the extrusion material measured at room temperature, and *L* represents the length when the material is heated to a specific temperature. The actuation stress is measured by a uniaxial tensile test instrument. The sample is fixed on the instrument and heated to a constant temperature with a hot-air gun. The surface temperature of the sample is measured by the infrared radiometer. The actuation performance of the printed LCE filaments under different processing conditions is shown in Figure 3. The actuation strain increases with the heating temperature. The contraction strain reaches the maximum value when heated to 150 °C. The length remains constant when the sample is further heated to higher temperatures. As shown in Figure 3A, the actuation stress and strain increase when the nozzle diameter decreases. The actuation stress of the printed LCE filament reaches 0.33 MPa at 150°C with a nozzle diameter of 0.6 mm. The printing height also significantly affects the actuation performance of the printed filaments (Figures 3C, D). The smaller the spacing, the greater the shrinkage and stress. According to Figures 3E, F, as the printing speed increases from 1 mm/s to 7 mm/s, the shrinkage of the printed LCE fibers increases and then decreases. At first, the actuation stress increases with the printing speed. It reaches a maximum value for the printing speed of 9 mm/s and then decreases. This can be attributed to a faster printing speed, which results in more LCEs being stretched by the nozzle and the substrate. Consequently, the alignment of the liquid crystal cells is improved. At relatively high printing speeds, the material does not have sufficient time to be fully extruded, which affects its shrinkage and actuation stress. The actuation force decreases with an increase in the printing speed. When the air pressure is constant, the printing speed is relatively fast, which decreases the fiber diameter since that part of LCE is too late to be extruded. As a result, the overall actuation force is smaller. The printing temperature significantly affects the contraction of LCE filaments (Figure 4A). The maximum contraction strain of the LCE filament with a nozzle diameter of 500 μm is 47.46% at 35°C, while the actuation strain printed at 75°C decreases to 2.86%. With a decrease in the printing temperature, the greater the shear and tensile action of the material during printing, the greater the actuation strain. When the printing temperature is too high, the material flow performance is enhanced, and shear and tensile actions are lowered. The relationship between the actuation strain and extrusion pressure is shown in Figure 4B. The overall contraction of the printed filaments increases with an increase in pressure. According to the contraction direction of the fiber, the rectangular film with length and widths of 20 and 5 mm was printed. Moreover, five sample films with a different number of layers were printed along the height direction. According to Figure 4C, the effect of the number of printing layers on the contraction strain of printed films is negligible.

### Deformation of 3D-printed LCE sample

**FIGURE 5:**
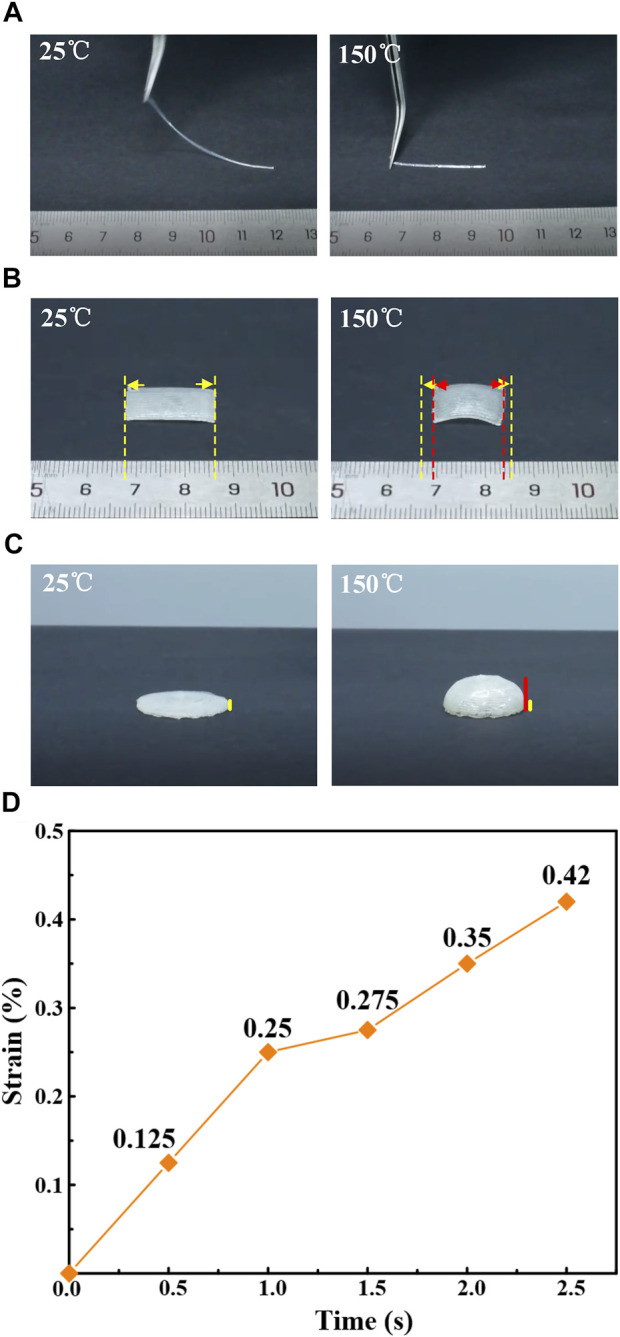
3D printed LCE sample structure and its actuation performance. The yellow line represents the original size, and the red line represents the deformed size. **(A)** LCE fiber shrinks along the length direction on the 150°C drying table. **(B)** LCE film contracts along the length direction on the 150°C drying table. **(C)** LCE film printed in circular coating mode shrinks and bulges towards the center (side view). **(D)** Variation of the actuation strain of the LCE fiber over time when heated on a 150°C drying table.

Some structures were printed (Figure 5) to investigate the effect of printing parameters on the actuation performance of LCE. The LCE was printed on the glass substrate in a fiber form (Figure 5A). At the normal temperature of 25°C, its original length is roughly 46.5 mm. When it is placed on the hot plate heated to 150°C, the sample contracts along the length direction, and the final contraction strain is approximately 42%. Variation in the contraction strain of LCE fiber with time on the hot plate heated to 150°C is shown in Figure 5D. The contraction strain of the sample is 12.5% in 1 s characterized by an upward trend, while all contraction is completed in 2.5 s. Similarly, a sample was printed in the form of thin film by linear coating, which had the same contraction along the moving direction of the printing nozzle on the hot plate at 150°C (Figure 5B). In addition, the printing process of moving from the outer circle to the inner circle at the same speed was programmed to obtain the circular coated sample. The deformation of the circular coated sample on the hot plate heated to 150°C is shown as a side view (Figure 5C). The circular sample gradually contracts and arches from the circumference to the center of the circle, the overall diameter decreases, and the side is shaped like a “hat”. Consequently, a double-layer rectangular LCE body was printed with a printing temperature of 40°C, printing height of 300 μm, extrusion pressure of 0.8 MPa, printing speed of 5 mm/s, and nozzle diameter of 600 μm.

### Fish-scale-like microstructures with different tilt angles and anisotropic performance testing

**FIGURE 6:**
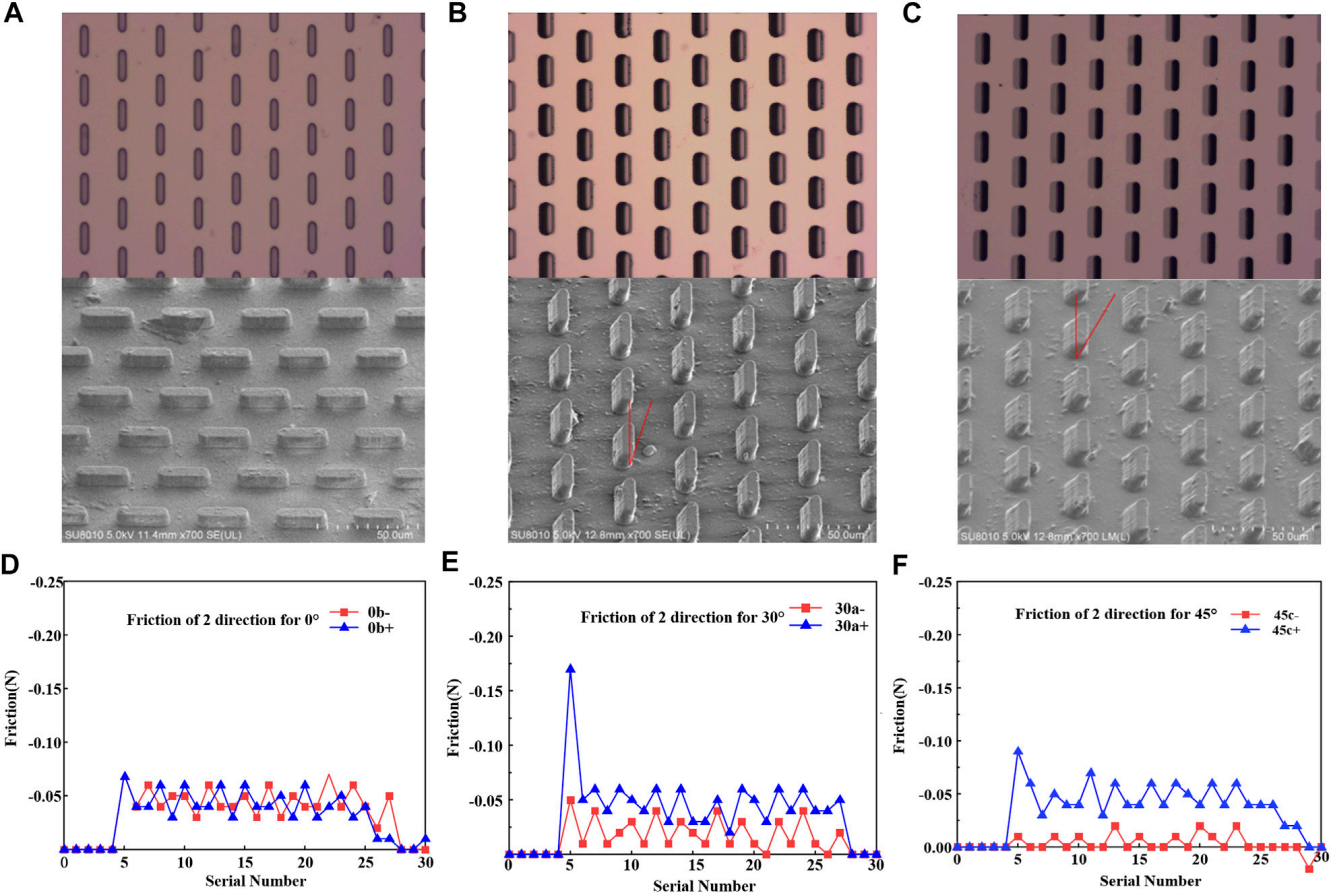
Images of the tilted fish-scale-like microstructures and their anisotropic friction curves at various angles. **(A–C)**: Top half of the figure: optical microscope diagram of the mold of the tilted fish-scale-like microstructures with inclined angles of 0°, 30°, and 45°; lower half of the figure: SEM images of the inclined fish-scale-like microstructures samples with inclined angles of 0°, 30°, and 45°. **(D–F)**: The friction force of the samples with inclined angles of 0°, 30°, and 45° when dragging along the flake structure (▲) and against the flake structure (■).

The tilted micropore structure of different inclined angles was obtained by adjusting the tilted angle of exposure, as shown in Figure 6. The upper half of 6A, B, and C represent the mold of tilted fish-scale-like microstructures. The bright long strip rectangle is the bottom part of inclined fish-scale-like microstructures, and the dark part is the side wall. Figures 6A–C depict molds with 0°, 30°, and 45° tilt angles, respectively. The inclined fish-scale-like microstructures PDMS structure are shown in the lower half of Figures 6A–C, from which it can be seen that the inclined angle successively increases from 0° to 30° and then to 45°.

The samples are pulled from two directions to test the anisotropic friction properties of the inclined fish-scale-like microstructures. Furthermore, the dynamometer is used to measure the dynamic friction force and static friction force of the inclined fish-scale-like microstructures when pulled and moved. The anisotropic performance can be demonstrated by analyzing the difference between the friction forces in the two directions.

The test bench mainly comprises a fixed slide block, moving slide block, dynamometer, cushion block, and lead screw guide rail. The prepared inclined fish-scale-like microstructures with inclination angles of 0°, 30°, and 45° were cut into the same size. The sample and the cushion block (the cushion block is used to make the force on the sample as horizontal as possible) are fixed on the fixed slider at one end, while the dynamometer is fixed on the moving slider at the other end. The movement is achieved by programming the stepping motor at one end of the lead screw.

The sample piece is bonded with the thin wire during the test via the adhesive tape. Then, one end of the thin wire is folded to the other end and bonded to the force-measuring head of the dynamometer. The motion parameters are programmed and set to ensure that the slider can move back and forth along the guide rail. The dynamometer pulls the sample to move for a certain distance during this process. The pull force measured by the dynamometer is the friction force between the sample and the moving surface during the movement of the sample. Moreover, 3 g weight is added to the sample to make the inclined fish-scale-like microstructures better contact the moving surface and more intuitively show the difference in friction values.

The test results are shown in Figures 6D–F. The drawing line of the sample dragged along the inclined direction of the fish-scale-like microstructures is marked as “▲”, and the drawing line of the sample dragged against the direction of the scale is marked as “■”. According to Figures 6D–F, with an increase in the inclination angle from 0° to 45°, the dynamic friction along the inclined direction of the microstructures fluctuates at approximately 0.05 N. However, the reverse friction decreases significantly from 0.05 N to roughly 0.01 N. Furthermore, as the tilt angle increases from 0° to 45°, the difference in friction between the two directions increases from nearly 0 N to approximately 0.04 N.

To summarize, it can be concluded that as the tilt angle of the scale structure increases from 0° to 45°, the friction difference between the two directions increases, and the performance of anisotropic friction is improved. It can be concluded that, as the inclination angle of the fish-scale-like microstructures increases from 0° to 45°, the friction difference in the two directions increases, and the performance of anisotropic friction is improved. The performance of anisotropic friction at 45° is the best among the three tilting angles. Therefore, the fish-scale-like microstructures with a tilting angle of 45° are chosen as the foot of the inchworm robot.

### The motion performance test of the robot

**FIGURE 7:**
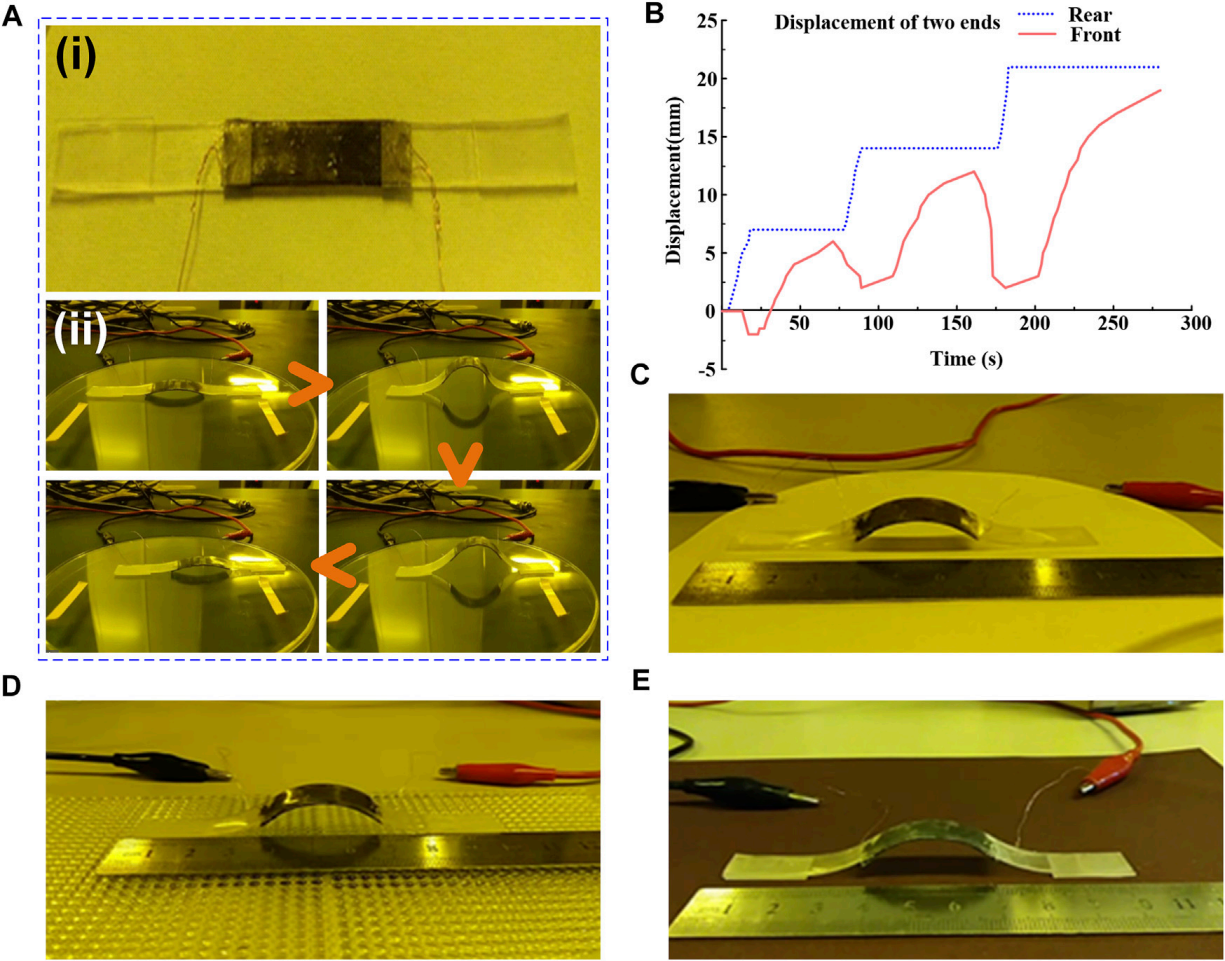
The crawling process and performance test of the inchworm-inspired soft robot. **(A)** Physical image of the robot and screenshots of the robot crawling on the Petri dish’s surface. **(B)** The displacement-time image of the robot crawling on the Petri dish’s surface. **(C)** Snapshot of the robot crawling on the surface of the filter paper. **(D)** Snapshot of the robot crawling on the surface of plexiglass with a convex spherical array **(E)** Snapshot of the robot crawling on the sandpaper surface.

The overall schematic diagram of the inchworm-inspired crawling soft robot is shown in Figure 1A. The thick solid black line is the glue bonding point. The final manufactured object of the crawling software robot is shown in Figure 7A(i).

The DC-regulated power supply is used as the power supply for the robot performance test. Firstly, a petri dish is selected as the platform for the crawling test. As shown in Figure 7A(ii), the inchworm-inspired crawling soft robot was placed on the Petri dish’s surface, where a double-sided adhesive was applied to determine the initial position for comparison. The red and black clips behind the petri dish are the positive and negative leads of the power supply, which are connected to both ends of the copper wire coming from the electrothermal film of the soft robot. The voltage used in this test is 30 V and the power on time is 30 s.

The video screenshot of the robot’s motion is shown in Figure 7A(ii). The robot bent and stretched for three cycles, spending a total of 4 min and 38 s and moving forward approximately 2 cm at a speed of roughly 4.3 mm/min. According to the diagram, it can be observed that the flexible actuator plays the role of actuation, and the feet at both ends show good anisotropy during movement, both of which keep the structure moving forward. It takes 30 s for the robot to achieve the arch but nearly 1 min to stretch. It is speculated that the long stretching time is due to the electrothermal film being wrapped by PDMS and LCE, which reduces the heat transfer between the film and the outside world. This is an area for improvement in this work. A displacement-time image of its movement on the top of the petri dish is drawn to represent the displacement of the inchworm-inspired robot more clearly, as shown in Figure 7B. The two curves in the figure represent the displacement changes of the front and rear feet with time. The rear foot represents anisotropic friction well, but the front end does not. It is believed that this is due to the change of force during the contraction of the flexible actuator, which affects its anisotropic frictional expression.

Next, to test the motion of the inchworm-inspired crawling soft robot on other surfaces, its movement on filter paper surface (Figure 7C), convex spherical array plexiglass surface (Figure 7D), and sandpaper surface (Figure 7E) are tested. The robot moves normally on all surfaces, proving that the inchworm-inspired crawling soft robot can move on different surfaces. In addition, it is found that the robot moves faster on the sandpaper surface and slower on the filter paper surface due to the different roughness, that is, different friction coefficients. However, the surface velocity of convex spherical array plexiglass is the slowest. This can be attributed to the structure that significantly undulates, and the inclined scale does not contact with it well. The dynamic motion of the soft robot on different surfaces is shown in Supplementary Video S1 in the supporting information.

## Discussion

In this study, the effects of 3D printing process parameters on the actuation performance of LCE were investigated in detail. Moreover, the optimized printing parameters were employed to make an LCE body and construct an inchworm-inspired soft robot. The electrothermal film was embedded in the body to actuate the robot’s motion. The anisotropic friction tilted fish-scale-like was employed to control the movement direction of the robot. The relationship between the anisotropy friction and the inclination angle of the scale was tested, and the best tilting angle was selected as 45°. A soft robot was assembled, and its motion performance on the Petri dish’s surface was tested under 30 V. The moving speed of the soft robot reached 4.3 mm/min. The moving performance on different surfaces was tested, which proves the universality of the bionic inchworm robot. The application of DIW technology in printable stimulation-response materials significantly contributed to achieving multi-functional and highly integrated soft robots, which opens a new avenue for the application of LCE in soft robotics.

## Data Availability

The original contributions presented in the study are included in the article/Supplementary Material, further inquiries can be directed to the corresponding author.
